# Neurons Putting Out the Trash: A Novel Facet of Proteostasis and Mitochondrial Quality Control

**DOI:** 10.1093/geroni/igaa057.2633

**Published:** 2020-12-16

**Authors:** Monica Driscoll

**Affiliations:** Rutgers University, Piscataway, New Jersey, United States

## Abstract

Toxicity of misfolded proteins and mitochondrial dysfunction are pivotal factors that promote age-associated functional neuronal decline and neurodegenerative disease across species. Although these neurotoxic challenges have long been considered to be cell-intrinsic, considerable evidence now supports that misfolded human disease proteins originating in one neuron can appear in neighboring cells, a phenomenon proposed to promote pathology spread in human neurodegenerative disease. We have found that C. elegans adult neurons that express aggregating proteins can extrude large (~5µM) membrane-surrounded vesicles that can include the aggregated proteins and damaged mitochondria. We speculate that throwing out the trash may correspond to a conserved mechanism that constitutes a fundamental, but formerly unrecognized, branch of neuronal proteostasis and mitochondrial quality control. I will discuss our current understanding of the mechanisms of neuronal trash elimination.

